# The superior predictive value of ^166^Ho-scout compared with ^99m^Tc-macroaggregated albumin prior to ^166^Ho-microspheres radioembolization in patients with liver metastases

**DOI:** 10.1007/s00259-019-04460-y

**Published:** 2019-08-09

**Authors:** Maarten L. J. Smits, Mathijs G. Dassen, Jip F. Prince, Arthur J. A. T. Braat, Casper Beijst, Rutger C. G. Bruijnen, Hugo W. A. M. de Jong, Marnix G. E. H. Lam

**Affiliations:** Department of Radiology and Nuclear Medicine, University Medical Center Utrecht, Heidelberglaan 100, 3584 CX Utrecht, The Netherlands

**Keywords:** Technetium-99m-MAA, Holmium-166 microspheres, Radioembolization, SIRT, Dosimetry

## Abstract

**Purpose:**

As an alternative to technetium-99m-macroaggregated albumin (^99m^Tc-MAA), a scout dose of holmium-166 (^166^Ho) microspheres can be used prior to ^166^Ho-radioembolization. The use of identical particles for pre-treatment and treatment procedures may improve the predictive value of pre-treatment analysis of distribution. The aim of this study was to analyze the agreement between ^166^Ho-scout and ^166^Ho-therapeutic dose in comparison with the agreement between ^99m^Tc-MAA and ^166^Ho-therapeutic dose.

**Methods:**

Two separate scout dose procedures were performed (^99m^Tc-MAA and ^166^Ho-scout) before treatment in 53 patients. First, qualitative assessment was performed by two blinded nuclear medicine physicians who visually rated the agreement between the ^99m^Tc-MAA, ^166^Ho-scout, and ^166^Ho-therapeutic dose SPECT-scans (i.e., all performed in the same patient) on a 5-point scale. Second, agreement was measured quantitatively by delineating lesions and normal liver on FDG-PET/CT. These volumes of interest (VOIs) were co-registered to the SPECT/CT images. The predicted absorbed doses (based on ^99m^Tc-MAA and ^166^Ho-scout) were compared with the actual absorbed dose on post-treatment SPECT.

**Results:**

A total of 23 procedures (71 lesions, 22 patients) were included for analysis. In the qualitative analysis, ^166^Ho-scout was superior with a median score of 4 vs. 2.5 for ^99m^Tc-MAA (*p* < 0.001). The quantitative analysis showed significantly narrower 95%-limits of agreement for ^166^Ho-scout in comparison with ^99m^Tc-MAA when evaluating lesion absorbed dose (− 90.3 and 105.3 Gy vs. − 164.1 and 197.0 Gy, respectively). Evaluation of normal liver absorbed dose did not show difference in agreement between both scout doses and ^166^Ho-therapeutic dose (− 2.9 and 5.5 Gy vs − 3.6 and 4.1 Gy for ^99m^Tc-MAA and ^166^Ho-scout, respectively).

**Conclusions:**

In this study, ^166^Ho-scout was shown to have a superior predictive value for intrahepatic distribution in comparison with ^99m^Tc-MAA.

## Introduction

Treatment planning for radioembolization (known as Selective Internal Radiation Therapy or SIRT) of liver tumors is generally performed with technetium-99m macroaggregated albumin particles (^99m^Tc-MAA). Aside from predicting the lung shunt and (other) non-target embolization, ^99m^Tc-MAA is also used to predict the activity distribution in the liver. Several studies have shown that the value of ^99m^Tc-MAA to predict the distribution of ^90^Y in the liver is limited [1–4]. Shape, size, density, and number of injected particles of ^99m^Tc-MAA differ greatly from ^90^Y-microspheres. This may explain the difference in biodistributions.

As an alternative to the yttrium-90 (^90^Y)-microspheres, microspheres containing the radioactive element holmium-166 (^166^Ho) have recently become available (Quiremspheres®, Quirem Medical, Deventer, the Netherlands). The radionuclide ^166^Ho provides several advantages over ^90^Y with regard to imaging possibilities. The element holmium itself is paramagnetic and can therefore be visualized—and quantified—on MRI [5, 6]. In addition, ^166^Ho emits low-energy gamma radiation, which can be used for quantitative SPECT/CT imaging [7]. A scout dose of ^166^Ho-microspheres (consisting of approximately 3 million microspheres with an activity of 250 MBq) can be used for treatment planning instead of ^99m^Tc-MAA [8]. This has the theoretical benefit of using the exact same ^166^Ho-microspheres for both procedures. The aim of the current study was to analyze whether the intrahepatic distribution of ^166^Ho-scout has a better agreement with the ^166^Ho-therapeutic dose distribution in comparison with ^99m^Tc-MAA.

## Materials and methods

### Patients and procedures

Patients treated with ^166^Ho-radioembolization in the phase 1 and phase 2 Holmium Embolization Particles for Arterial Radiotherapy (HEPAR) studies were analyzed (Clinicaltrials.gov numbers NCT01031784 and NCT01612325). All patients had unresectable liver metastases from various primaries. The institutional review board approved these studies and all patients provided written informed consent. All patients received treatment planning with the conventional ^99m^Tc-MAA and subsequently with a scout dose of 250 MBq of ^166^Ho-microspheres (60 mg; approximately 3 million microspheres). Patients were included for analysis if they had received both pre-treatment administrations of ^99m^Tc-MAA and ^166^Ho-scout before treatment with ^166^Ho-microspheres.

All radioembolization procedures were performed according to the HEPAR study protocol [9]. Non-target vessels were only coil-embolized in case no safe injection position could be found distal to these non-target vessels. Approximately 150 MBq ^99m^Tc-MAA (0.8 mg, approximately 1.8 million particles, TechneScan LyoMAA; Mallinckrodt Medical B.V., Petten, the Netherlands) was injected. ^99m^Tc-MAA was injected slowly at approximately 5 ml/min. All injections (both ^99m^Tc-MAA and ^166^Ho-microspheres) were performed with a standard 2.4F or 2.7F microcatheter (Progreat®, Terumo, Japan). At the end of the procedure, the access site in the groin was compressed for hemostasis. ^99m^Tc-MAA injection was followed by planar- and SPECT/CT imaging to check for extrahepatic deposition and excessive lung shunting (> 30 Gy lung absorbed dose). If no contra-indications were found, the patient was scheduled for treatment. In case the ^99m^Tc-MAA procedure had to be repeated because of extrahepatic activity, only the most recent ^99m^Tc-MAA data were used for analysis. No vessels were coil-embolized after the final ^99m^Tc-MAA procedures. On the day of treatment, patients underwent a second treatment-planning procedure in the morning. During this procedure, the injection position(s) from the first treatment planning procedure was mimicked and patients received a scout dose of 250 MBq of ^166^Ho-microspheres (approximately 3 million microspheres). At the end of the procedure, the catheter and microcatheter were removed. The vascular sheath was left in the groin in order to secure vascular access for the treatment procedure in the afternoon. The sheath was connected to a pressurized bag of saline (for continuous flushing) and secured with sterile tape. This second treatment-planning procedure was again followed by planar imaging and SPECT/CT. If there were no contra-indications for SIRT, patients returned to the angio-suite in the afternoon to receive treatment with ^166^Ho-microspheres. Catheters were introduced via the vascular sheath that had remained in situ. Again, injection position(s) of the treatment-planning procedures were mimicked. Total treatment activity was planned based on an aimed whole liver absorbed dose of 20, 40, 60, and 80 Gy for patients who were treated in the phase 1 HEPAR study and 60 Gy for patients in the phase 2 HEPAR study [10, 11]. The total number of microspheres injected was the same for all patients (i.e., 600 mg; approximately 30 million microspheres). After injection, the catheters and sheath were removed and a vascular closure device was used for hemostasis.

In order to exclude cases in which differences in injection positions might have influenced distribution, agreement of catheter-tip positions between the three procedures (i.e., ^99m^Tc-MAA, ^166^Ho-scout, and ^166^Ho-therapeutic dose) was retrospectively analyzed by three observers (M.S, A.B., and M.L.). These observers independently reviewed the agreement of all injection positions per procedure on digital subtraction angiography images. Agreement was rated on a 4-point scale (1, very poor agreement, difference in catheter tip position > 10 mm; 2, poor agreement, difference 5–10 mm; 3, good agreement, difference 3–5 mm; 4, very good agreement, difference < 3 mm). Only the patients with good or very good agreement (point scale 3 and 4) between all the injection positions were included in this study.

In case of procedures with multiple injection positions, the ratio between the injected activities at each injection position had to be the same for the three procedures. This ratio was based on CT-volumetry (e.g., a 2:1 activity ratio for a right liver lobe of 1000 mL and a left liver lobe of 500 mL). Agreement between the procedures was checked based on the administered activity per injection position, which was corrected for any residual activity. A maximum deviation of 10% between the two pre-treatment and post-treatment injections was accepted.

### Imaging

Pre- and posttreatment SPECT/CT imaging was performed on a dual-headed gamma camera (Forte, Philips Medical Systems, 6 procedures) and a SPECT/CT camera (Symbia 16T, Siemens Health Care, 17 procedures). In all cases, the same scanner was used for pre- and posttreatment SPECT/CT. Pre-treatment ^99m^Tc-MAA SPECT images were acquired on a 512 × 512 matrix (15 procedures) and a 128 × 128 matrix (8 procedures). An energy window of 129.1- to 150.5-keV and a low-energy general-purpose collimator were used. Pre- and post-treatment ^166^Ho SPECT images (interval; mean 4 days, range 3–6 days) were acquired on a 128 × 128 matrix (22 procedures) and a 512 × 512 matrix (1 procedure) in combination with a 74.9- to 87.1-keV energy window and a medium-energy general-purpose collimator. Imaging was performed with 120 projections over a noncircular orbit of 360° (^99m^Tc-MAA: 30s/projection (Philips) or 20s/projection (Siemens) and ^166^Ho; 30s/projection). An in-house developed and validated Monte-Carlo-based reconstruction algorithm (Utrecht Monte-Carlo System (UMCS)) intrinsically correcting for attenuation and scatter was used for the reconstruction of the ^99m^Tc-MAA and ^166^Ho HEPAR I data [12]. The reconstruction of all HEPAR II data was done by using the Siemens Flash3D ordered-subsets expectation maximization (^99m^Tc-MAA; 6 iterations, 8 subsets, ^166^Ho; 5 iterations 8 subsets). The scatter correction for the HEPAR II ^99m^Tc-MAA data was applied by using the dual energy window method with two adjacent energy windows (both 15% width) and a lower window weight of 0.5. The HEPAR II ^166^Ho data was reconstructed without scatter correction. For ^99m^Tc-MAA data, a Gaussian post-reconstruction filter of 5 mm in full width at half maximum was also included. Reconstructed voxel sizes were 4.66 × 4.66 × 4.66 mm^3^ for all Philips images and in the range of 0.70 × 0.70 × 4.03 mm^3^ to 3.90 × 3.90 × 4.03 mm^3^ for the Siemens ^99m^Tc-MAA and 4.80 × 4.80 × 4.80 mm^3^ for the Siemens ^166^Ho images, respectively.

### Qualitative analysis

Two nuclear medicine physicians independently compared the intrahepatic distribution of activity of ^99m^Tc-MAA and ^166^Ho-scout with the post-treatment ^166^Ho-therapeutic dose. Image sets of all three SPECT scans were coupled, blinded, and simultaneously presented to the reviewers in our Picture Archive and Communication System (Sectra PACS, Almere, the Netherlands). The post-treatment SPECT was marked “post-treatment” to allow the reviewers to compare it with the other two pre-treatment SPECT scans (which were marked “pre-treatment”). No information was provided regarding the type of pre-treatment scan. To prevent bias, both pre-treatment scans were placed randomly on the screen for each patient. Visual agreement was rated on a 5-point scale (1–5, very poor agreement–very good agreement).

### Quantitative analysis

**Fig. 1:**
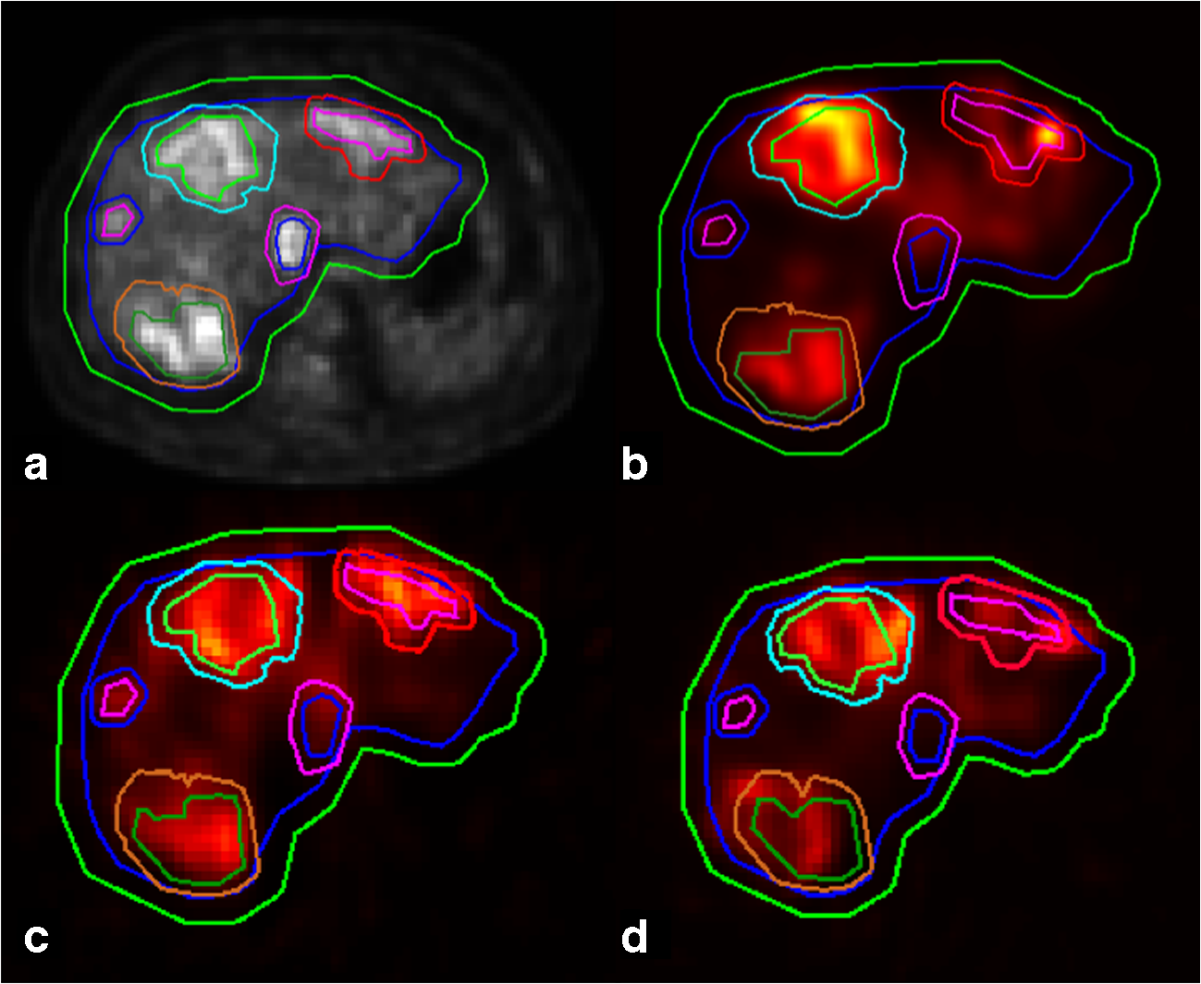
Example of liver and tumor segmentation on FDG-PET (**a**) and co-registration on ^99m^Tc-MAA (**b**), ^166^Ho-scout (**c**), and ^166^Ho-therapeutic dose (**d**) SPECT images. The delineated VOIs were extended with a margin of 1 cm; therefore, each target volume is surrounded by a second line

Agreement of intrahepatic distribution was also measured quantitatively. FDG-PET/CT pre-treatment scans were used for segmentation of lesion and liver volumes (volumes of interest, or VOIs). In-house-developed software was used for this purpose (VolumeTool, version 1.6.5.) [13]. Delineation of VOIs was performed manually on FDG-PET images. The normal liver was defined as the whole liver minus tumors. All VOIs were manually registered (non-deformable) to the ^99m^Tc-MAA SPECT, ^166^Ho-scout SPECT, and ^166^Ho-therapeutic dose SPECT images (Fig. 1). In order to compensate for co-registration errors and blurring effects, the VOIs were enlarged by a 1-cm margin when measuring the activity. Also, small lesion VOIs with a volume smaller than 10 mL were excluded, because of their higher sensitivity to these errors. The pre-treatment ^99m^Tc-MAA SPECT and ^166^Ho-scout SPECT and the post-treatment ^166^Ho-therapeutic dose SPECT were converted into units of ^166^Ho-activity concentration by normalization of the total number of reconstructed counts to the total administered activity of ^166^Ho during therapy. This resulted in two predictive amounts of ^166^Ho (MBq/cm^3^) for each VOI, based on both pre-treatment SPECT images, in comparison with an actual amount of ^166^Ho based on post-treatment SPECT. The lesion-absorbed dose was calculated by dividing the activity measured in the lesion VOI plus the 1-cm margin by the volume of that same lesion VOI without the 1-cm margin. Normal liver activity was calculated by subtraction of the measured activity of all lesion VOIs within one patient (including the VOI’s with a volume smaller than 10 mL) without 1-cm margin from the activity of the liver VOI with 1-cm margin. The volume of the normal liver compartment was calculated by subtraction of the total volume of the lesion VOIs without margin from the volume of the liver without margin. Finally, the calculated activity was divided by this volume to calculate the normal liver absorbed dose. No extrahepatic activity distribution and no interval change in liver morphology were assumed. Contribution of gamma radiation was neglected in calculating the absorbed dose. To illustrate clinical implications, the calculated activity concentrations were converted into units of Gray for both the lesion and normal liver segmentations, using a conversion factor of 15.78 ∙ 10^−3^ Gy/(MBq/kg) assuming a liver density of 1.06 kg/L [10].

### Statistical analysis

Statistical data analysis was performed using a commercial statistical software package (SPSS for Windows, version 21.0; SPSS Inc.). For the qualitative analysis, medians and interquartile ranges were calculated and the Wilcoxon signed rank test was used to analyze differences in overall agreement scores between both pre-treatment scans and the ^166^Ho treatment scan. The Weighted Kappa (squared) was calculated to classify the interrater agreement [14, 15].

Bland-Altman plots were used for evaluating agreement between pre- and post-treatment activity biodistributions [16, 17]. The confidence interval of the 95% limits of agreement was used to test the significance of the difference in agreement for both plots. The precision of the 95% limits of agreement was estimated by the number of analyzed procedures or lesions (*n*), the standard deviation of the differences (*s*), and the degrees of freedom (*n* − 1). In this estimation, no corrections were made for any correlation between analyzed lesions within a patient. The 95% confidence intervals were calculated by the multiplication of the standard error (standard error = $\sqrt{3{\mathrm{s}}^2/n}$) of the upper and lower 95% limit of agreement and the value of the *t* distribution with *n* − 1 degrees of freedom according to the method described by Bland and Altmann [16]. Agreement was considered statistically significantly different if the bandwidth of the 95% limits of agreement, including the confidence intervals, of either scout method was smaller than the bandwidth of the other scout method.

## Results

**Fig. 2:**
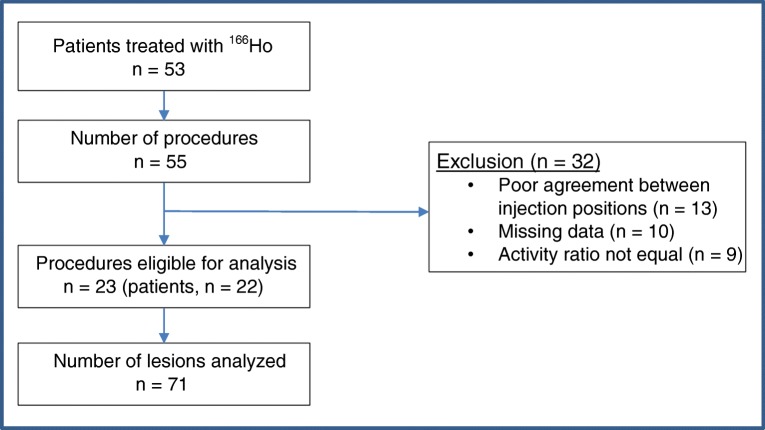
Inclusion flowchart of patient data

Fifty-three patients underwent 55 treatment procedures between December 2009 and March 2015 (two patients underwent separate lobar procedures) (Fig. 2). Before treatment, every patient received ^99m^Tc-MAA and ^166^Ho-scout in a separate procedure (^99m^Tc-MAA interval; median 7 days, range 2–21 days and ^166^Ho-scout; same day). Thirty-two procedures were excluded from analysis: in 13 procedures, a mismatch in catheter tip positioning was found; in 10 procedures, data was not completely available (one or more missing angiographic images (*n* = 4); no pre-treatment FDG-PET/CT (*n* = 2); no post-treatment SPECT/CT (*n* = 2); ^166^Ho-scout was not administered (*n* = 2)), and in nine procedures, the ratio of activity between the injection positions was not similar. A total of 23 procedures in 22 patients were included for analysis (Table 1). In these 22 patients, 71 lesions were analyzed. The mean administered treatment activity of ^166^Ho-microspheres per procedure was 5,470 MBq (range 1,957–12,897).

**Table 1:** Baseline characteristics

Characteristic	Patients, (*n*)
Sex, male/female	16/6
Age, year: median (range)	62.1 (38–84)
Primary tumor	
Colorectal carcinoma	11 (50%)
Uveal melanoma	5 (23%)
Cholangiocarcinoma	2 (9%)
Mamma carcinoma	1 (5%)
Other *	3 (14%)
^166^Ho-microspheres activity in MBq: mean (range)	5470 (1,957–12,897)
^99m^Tc-MAA activity in MBq: median (range)	145 (65–180)
^166^Ho-scout activity in MBq: median (range)	258 (103–292)
^99m^Tc-MAA lung shunt fraction in %: mean (range)	5.6 (1.0–13.4)
Liver tumor involvement	
< 25%	17 (77%)
25–50%	4 (18%)
50–75%	1 (5%)
75–100%	0 (0%)
Treatment	
Whole liver in one procedure	20 (91%)
Whole liver in two procedures	1 (5%)
Lobar right only	1 (5%)
Lobar left only	0 (0%)
Injection positions per procedure	
1 position	10 (43%)
2 positions	11 (48%)
3 positions	2 (9%)
Total included procedures	23
Total included lesions	71
Lesion volume in mL: median (range)	36 (10–1,598)
Previous liver-directed treatment	
Partial liver resection	4 (18%)
Radiofrequency ablation (RFA)	1 (5%)
External beam radiation	1 (5%)
Radioembolization	0 (0%)
Previous systemic treatment	21 (95%)

*Neuroendocrine (1); pancreatic (1); gastric (1)

**Fig. 3:**
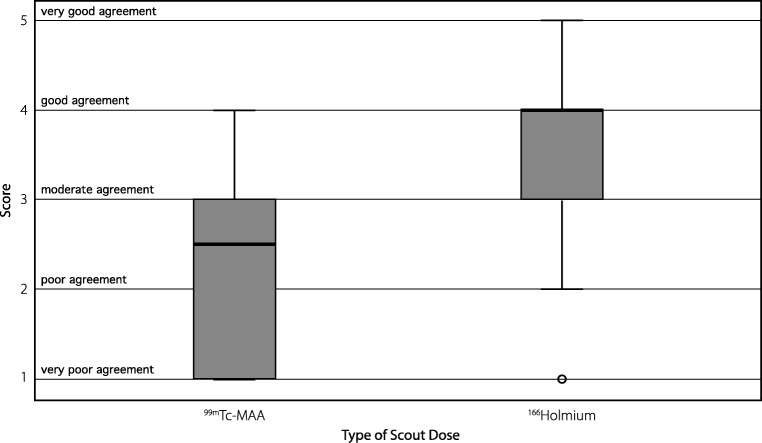
Box plot. Scores of the overall agreement between both ^99m^Tc-MAA and ^166^Ho-scout and ^166^Ho-therapeutic dose of all 23 procedures are plotted in a box plot. Median and interquartile lines are indicated. Holmium scout dose performs significantly better than ^99m^Tc-MAA (*p* < 0.001)

**Fig. 4:**
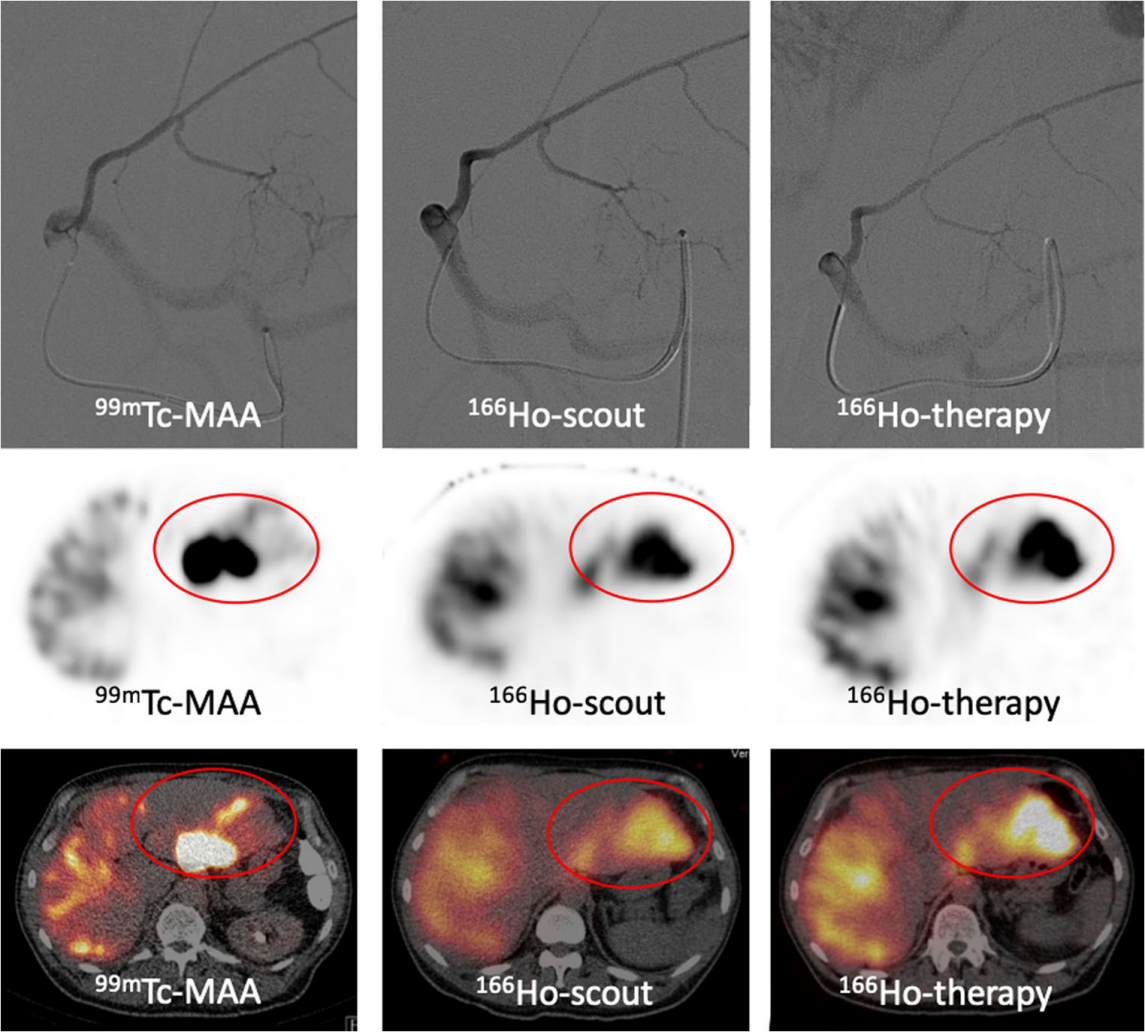
Example of discrepancy between ^99m^Tc-MAA and ^166^Ho-therapeutic dose. Despite identical catheter positions (upper row), there is a remarkable difference in activity distribution between the three procedures. SPECT-CT (middle and lower row) shows that the activity distribution in the liver of ^166^Ho-scout is more similar to the therapy distribution than ^99m^Tc-MAA. Overall agreement of ^99m^Tc-MAA was rated 2.5 compared to 4.5 for ^166^Ho scout for this patient

Figure 3 shows the overall agreement scores of both pre-treatment scans with the ^166^Ho post-treatment scan, based on the visual assessment of the two nuclear medicine physicians. The median score for ^99m^Tc-MAA was 2.5 compared to 4 for ^166^Ho-scout (*p* < 0.001). In 15 of the 23 procedures, overall agreement between ^166^Ho-scout and ^166^Ho-therapeutic dose was rated higher than ^99m^Tc-MAA (Fig. 4). ^99m^Tc-MAA showed better overall agreement in five procedures and in three procedures equal agreement was observed. The interrater agreement was moderate with a weighted kappa of 0.52.

**Fig. 5:**
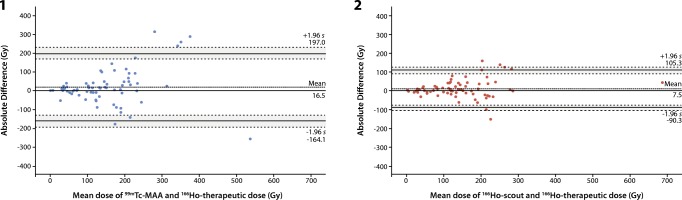
Bland-Altman plots for lesion analyses. (1) Difference between ^99m^Tc-MAA and ^166^Ho-therapeutic dose activity in each lesion is plotted against mean activity in each lesion. (2) Difference between ^166^Ho-scout and ^166^Ho-therapeutic dose activity in each lesion is plotted against mean activity in each lesion. The 95% limits of agreement (LoA) are indicated and surrounded by two dotted lines indicating the standard error of the 95% LoA

Quantitative analysis showed that the overall mean differences in pre- minus post-treatment calculated absorbed dose for all lesions were 16.5 Gy for ^99m^Tc-MAA and 7.5 Gy for ^166^Ho-scout. For ^99m^Tc-MAA the 95% limits of agreement of the differences were − 164.1 and 197.0 Gy (Fig. 5). ^166^Ho-scout showed 95% limits of agreement of the differences of − 90.3 and 105.3 Gy. Based on the narrower width of the 95% limits of agreement and no overlap in confidence intervals of the 95% limits of agreement of the ^99m^Tc-MAA (− 197.3 and − 130.8 Gy and 163.8 and 230.3 Gy) and ^166^Ho-scout (− 108.3 and − 72.3 Gy and 87.3 and 123.4 Gy) Bland-Altman plots, ^166^Ho scout performed significantly better than ^99m^Tc-MAA.

**Fig. 6:**
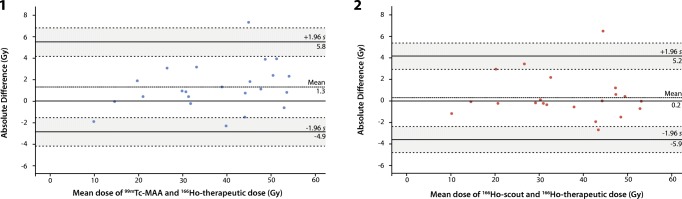
Bland-Altman plots for healthy-liver segmentation. (1) Difference between ^99m^Tc-MAA and ^166^Ho-therapeutic dose activity is plotted against mean activity for each procedure. (2) Difference between ^166^Ho-scout and ^166^Ho-therapeutic dose activity is plotted against mean activity for each procedure. The 95% limits of agreement (LoA) are indicated and surrounded by two dotted lines indicating the standard error of the 95% LoA

Quantitative analysis of the normal liver absorbed dose showed that the mean difference in the pre- and post-treatment calculated absorbed dose was 1.3 Gy for ^99m^Tc-MAA and 0.2 Gy for ^166^Ho-scout (Fig. 6). The 95% limits of agreement of ^99m^Tc-MAA and ^166^Ho-scout were comparable, − 2.9 and 5.5 Gy for ^99m^Tc-MAA and − 3.6 and 4.1 Gy for ^166^Ho-scout with overlapping confidence intervals (^99m^Tc-MAA; − 4.2 and − 1.6 Gy and 4.1 and 6.8 Gy, ^166^Ho-scout; − 4.9 and − 2.4 Gy and 2.9 and 5.3 Gy).

## Discussion

This study showed that treatment planning prior to radioembolization can be improved by using a scout dose of ^166^Ho-microspheres instead of ^99m^Tc-MAA. The qualitative and quantitative analysis showed that the agreement between ^166^Ho-scout and ^166^Ho-therapeutic dose was significantly superior to ^99m^Tc-MAA.

The difference in agreement between scout dose distribution and treatment distribution is subject to several influencing factors. First, as stated before, catheter positioning is known to be a key factor [2]. In order to accurately measure agreement, the catheter tip should be positioned identically for all administrations in each patient to prevent differences in flow. Attempts were made to eliminate this factor by always paying close attention to the exact positioning of the catheter and by retrospectively excluding patients for whom the catheter positions showed suboptimal agreement. However, even with seemingly identical catheter positions on 2D images, the catheter position in 3D may still be different.

Secondly, the assumption of a homogeneous distribution in the normal liver and lesion segmentations is not representative, leading to an overestimation of agreement. From a clinical perspective, normal liver-absorbed dose and tumor-absorbed dose are the most important parameters in terms of safety and efficacy. However, the VOIs of the normal liver compartment cover a much larger volume than the lesion VOI’s and therefore the level of overestimation of the agreement will be higher for this part of the analysis. In the qualitative analysis, agreement was visually analyzed on a more detailed, sub-segmental level. Interestingly, this analysis showed that in 65% of the 23 procedures, overall agreement between ^166^Ho-scout and ^166^Ho-therapeutic dose was better than with ^99m^Tc-MAA. In addition, the mean of the overall agreement scores of all procedures of ^166^Ho-scout was significantly higher compared to ^99m^Tc-MAA.

Thirdly, the width of the standard error of the 95% limits of agreement is strongly influenced by the number of analyzed procedures. Due to strict exclusion criteria, only 23 procedures were eligible for analysis. A larger study would require more patients to undergo two types of treatment-planning administrations, which is a burden to the patients, costly and arguably unethical.

Lastly, technical aspects may have influenced the outcome of this study. Imaging of ^166^Ho and ^99m^Tc-MAA is inherently different in terms of background noise and resolution, which could have influenced both the qualitative and quantitative analyses. These inherent differences are unavoidable for conducting this study. In addition, the type of gamma camera, reconstruction software and matrix size was not uniform across the study. We think the influence of these factors on the outcome of the analyses is limited. A more important factor is the interval between injections. The injections of ^166^Ho-scout and ^166^Ho-therapeutic dose were performed on the same day. It is possible that the scout dose injection induced embolic effects that influenced the distribution of ^166^Ho-microspheres during the treatment injection later that day. The majority of the analyzed patients were treated with radioembolization for colorectal liver metastases that are known to be relatively hypovascular tumors. Especially in these patients, embolic effects may lead to alterations in microsphere distribution [18]. This may have led to an enhanced disagreement between ^166^Ho-scout and ^166^Ho-therapeutic dose. Regardless of the type of scout-dose, embolic effects can be experienced during ^166^Ho-radioembolization, even though the number of ^166^Ho-microspheres injected for therapy (approximately 30 million) is lower than the typical number of resin ^90^Y-microspheres injected (estimated 50 million) [9, 11]. On the other hand, however, the larger time interval between ^99m^Tc-MAA (i.e., approximately 1 week versus same day for ^166^Ho-scout) and ^166^Ho-therapeutic dose may have led to interval changes in flow dynamics and thus altered distribution. Any type of scout dose performed on the same day as treatment may be more predictive than scout doses injected earlier. The difference in time interval may also result in ^99m^Tc-MAA being administered by another operator than the operator injecting the ^166^Ho-therapeutic dose. Administration speed and technique may differ between operators. Also, the administration box used for ^166^Ho-scout and therapy administration with the possibility to intermittently change between contrast and microsphere injections was not available for ^99m^Tc-MAA injections.

A downside of ^166^Ho-scout is that it is not as readily available as ^99m^Tc-MAA, which can be easily extracted from a generator. ^166^Ho-scout needs to be ordered, activated in a nuclear reactor and delivered to the treatment site.

This study focused on intrahepatic distribution, but lung shunt calculation is another important aspect of ^166^Ho-scout. Elschot et al. showed that a scout dose of ^166^Ho-microspheres is more accurate for lung shunt calculation on SPECT/CT than ^99m^Tc-MAA [19]. This reduces the chance that a patient is unnecessarily excluded from therapy or receives an unnecessary dose reduction. In addition, Braat et al. studied safety concerns of an unintended extrahepatic deposition of ^166^Ho-scout. They showed that extrahepatic depositions did not lead to any adverse events in their study. This supported the previously published findings by Prince et al. [8, 20].

Individualized treatment planning becomes increasingly important for patients who are selected for radioembolization. However, pre-treatment activity planning based on ^99m^Tc-MAA lacks dosimetric accuracy and needs improvement in order to increase the clinical benefit of radioembolization treatment. A recent study by Kafrouni et al. showed that body surface area (BSA) based activity planning (i.e., the “BSA-method”) often leads to under-dosing, most likely because it insufficiently corrects for the tumor-to-normal (T/N) uptake ratios [21]. Despite this limitation, the BSA-method currently is the most commonly used treatment planning method [22]. As an alternative to the BSA-method, the so-called partition model can be used. Analysis of SPECT-based biodistribution of ^99m^Tc-MAA is used to account for distribution differences between tumor and non-tumor compartments. This method is known to be a more personalized and accurate treatment planning method. However, the predictive value of ^99m^Tc-MAA seems limited in many cases and the partition model is rarely used in clinical practice. The therapeutic activity choice in our treatment is based on one-compartment modeling or the so-called MIRD model, using an average absorbed dose to the target volume without differentiation between tumor and normal tissue absorbed doses. Analysis of ^166^Ho-scout and ^166^Ho-therapeutic dose imaging, together with treatment outcomes in terms of efficacy and safety, will lead to threshold values for personalized dosimetry-based treatment planning, which ultimately needs to be validated in prospective clinical studies. This study showed that ^166^Ho-scout may serve as a predictive “biomarker” for safe and effective treatment.

In conclusion, both the qualitative and quantitative analyses showed that the intrahepatic distribution of the ^166^Ho-scout agreed better with the distribution of the therapy dose than ^99m^Tc-MAA, although the comparison has some inherent limitations. These results support the use of a scout dose of ^166^Ho-microspheres for radioembolization treatment planning.
